# How Do French Parents Determine Portion Sizes for Their Pre-Schooler? A Qualitative Exploration of the Parent–Child Division of Responsibility and Influencing Factors

**DOI:** 10.3390/nu13082769

**Published:** 2021-08-12

**Authors:** Kaat Philippe, Sylvie Issanchou, Anaïs Roger, Valérie Feyen, Sandrine Monnery-Patris

**Affiliations:** Centre des Sciences du Goût et de l’Alimentation, AgroSup Dijon, CNRS, INRAE, Université Bourgogne Franche-Comté, 21000 Dijon, France; sylvie.issanchou@inrae.fr (S.I.); anais.roger@inrae.fr (A.R.); valerie.feyen@inrae.fr (V.F.); sandrine.monnery-patris@inrae.fr (S.M.-P.)

**Keywords:** qualitative research, food portioning practices, children, parental feeding practices, food culture, autonomy

## Abstract

Large portion sizes can make children overeat, alter their self-regulation abilities and induce weight gain. However, little is known about how parents determine portion sizes for their children. Using semi-structured interviews with 5 fathers and 32 mothers of pre-schoolers, this study examined French parents’ food portioning practices. The division of responsibility between parent and child in deciding portion sizes was explored, as well as the influencing factors and possible sources of information. Parents described a wide range of practices. For most, determining portion sizes is an intuitive action that depends on habits and mainly arises from experiences with feeding their child and his/her appetitive traits. Few parents grant autonomy to their child for portioning and serving food, especially for the first serving. Many influencing factors were identified, including child-related (e.g., appetite, food preferences), parent-related (e.g., avoiding food waste), and external factors (e.g., influence of siblings, French food culture). Most parents do not search for information/recommendations to guide their practices. Stimulating optimal self-regulation of eating in children is important and parents can play a crucial role in this. This study identified barriers and facilitators to guide parents in providing appropriate portion sizes and help include children in this decision process.

## 1. Introduction

Parents play a key role in the development of young children’s eating habits and preferences [1]. Parental feeding practices, or the behavioral strategies used to control what, how much, when, and where the child eats [2], constitute a possible means to prevent the emergence of “unhealthy” eating habits and obesity. According to the theory of division of responsibility in feeding [3], parents should be primarily responsible for the choice of foods served to the child (what), as well as where to and when to eat, while the child should decide on how much food to eat and whether to eat. This is based on the idea that children have the capacity to self-regulate their food intake according to their physiological needs, managed by their sensations of hunger and fullness [4]. In order to maintain optimal self-regulation of intake, the feeding relationship must be supportive and responsive to the child’s needs and capacities [5,6]. There must be both autonomy granted to the child and leadership taken up by the parent—for example, by providing a social context for feeding and enforcing certain rules and boundaries.

In practice, however, parents do not seem to follow these recommendations; they tend to grant their child too much autonomy for deciding what to eat, but too little autonomy for deciding how much to eat [7]. It has been reported that if children rely on environmental factors for their intake (e.g., inappropriate portion sizes, controlling parental feeding practices, availability of palatable foods) rather than their internal signals of hunger and fullness, this could cause them to overeat, which in turn, if applied consistently, could result in an increased risk of overweight and obesity [8,9,10,11,12]. The “portion size effect” can illustrate this risk: when humans are served larger portions, they tend to eat more, and this has been demonstrated robustly and reliably in both adults (for a review, see [13]) and children (for a review, see [14]). A meta-analytic review by Zlatevska et al. [15] indicated that when a double portion size is served, energy intake in adults and children increases by 35% on average.

In addition, since young children are highly dependent on their parents for their food intake, it is important to gain additional insight into parental food portioning practices, the division of autonomy between parent and child in terms of determining portion sizes, and the drivers of these practices. Here, the term “parental portioning practices” refers to the decisions made by parents regarding the portion sizes of foods or beverages they serve to their child. In 2018, Kairey et al. [16] published a systematic literature review on this topic, including 14 quantitative and 14 qualitative studies with parents of children aged 2–12 years. The results of this review provide valuable insight into parental food portioning practices and the drivers of these practices, but it is important to note that the majority of the included studies were conducted in the USA (quantitative 11/14, qualitative 10/14). Knowing that cultural differences exist with regard to eating habits, food attitudes [17,18], and parental feeding practices [19,20], these results may not be automatically generalized to other countries and cultures. To our knowledge, no qualitative study has examined parental food portioning practices for pre-schoolers and the factors influencing them in France. The specificity of the meal structure in France, where lunch and dinner are usually composed of different components (i.e., starter, main course, dairy product, dessert) could, for example, give rise to specific parental portioning practices.

Therefore, the main aim of the present study is to capture the variety of parental portioning practices used for French pre-schoolers. Following the idea of Satter’s theory of division of responsibility [3], the degree of autonomy granted to the child for serving food and determining portion sizes will be explored here. The current study is explorative, but we expect to observe a wide range of parental portioning practices, as well as differences in the degree of autonomy granted to children in terms of deciding how much to eat. As observed by Loth et al. [7], we expect most parents to grant their child little autonomy in deciding how much to eat. 

The second aim of this study is to identify the factors that underlie parental portioning practices (e.g., parental perceptions, cultural aspects). Finally, the third aim of this study is to explore parental use of information sources and recommendations regarding the determination of portion sizes and parents’ perceptions and expectations about their use. This could be useful for identifying parents’ need for guidance. 

In the present study, parents of pre-schoolers aged 3 to 5 years will be targeted, as this period can be challenging due to the peak in children rejecting food at this age [21,22] and the deterioration in children’s self-regulation of food intake [8,23]. Furthermore, parents with different educational levels and different family situations (two-parent vs. single-parent households, different birth order of the target child) will be included because it is known that parental practices can vary according to these characteristics. For example, a recent cross-sectional study in France [24] using questionnaires found that mothers with a lower level of education served larger portions to their child aged 8–11 years than mothers with a higher level of education.

## 2. Materials and Methods

### 2.1. Ethics Statement

Ethical approval (n°20-686) was granted for this study by the Institutional Review Board (IRB00003888, IORG0003254, FWA00005831) of the French Institute of Medical Research and Health. Study registration was conducted by the data protection service involved (CNRS).

### 2.2. Participants

Parents were eligible to participate if they were older than 18 years and if they had at least one child aged 3–5 years. They were not eligible if their child suffered from a condition that could influence his/her eating (e.g., swallowing difficulties, autism), as we estimated that this could impact the feeding interactions between parent and child. Parents were recruited from a research panel in Dijon (ChemoSens Platform’s PanelSens, CNIL no. 1148039) via schools and via snowball sampling. A sampling matrix was used to select a sample of participants who were diverse in terms of parental sex, level of education, household composition, and birth order of the child. When conceptualizing this study, it was estimated that approximately 40 parents would be needed to ensure that there was a diverse sample of participants. However, a possible “saturation” effect of the data was evaluated during the data collection phase, meaning that the recruitment of a certain group of participants would be stopped if new interviews did not provide additional information to that found in previous interviews of this group.

Finally, a total of 37 parents (32 mothers, 5 fathers) were selected to participate in this study. Their characteristics and those of their children are presented in Table 1. Parents received a voucher of twenty Euros to thank them for their participation.

### 2.3. Data Collection

Data collection took place in summer 2020, right after the first COVID-19 lockdown in France. Due to ongoing infection risk at the time of data collection, it was not possible to conduct the planned interviews in person. All steps of data collection were therefore adapted so that they could be performed remotely, without physical presence.

#### 2.3.1. Recruitment form and Informed Consent

Parents who were interested in participating were invited to complete an online recruitment form. In this form, they were asked to provide information about their sex, age, level of education, and household composition, as well as about their child’s age, birth order, and possible illnesses influencing his/her eating behavior. Parents were also asked to state their availability for a possible interview and to provide their contact details. 

When parents were selected to participate, they received a link to an online informed consent form. Information about this study and the data protection policy was provided here. Parents were asked to confirm that they understood and agreed with the information provided and that they agreed to have the interview recorded.

#### 2.3.2. Interview Guide and Interview Procedure

Semi-structured telephone interviews were conducted in parents’ native language (French). Interviews lasted an average of 43 min (from 23 to 78 min). They were conducted by a research engineer, a research technician and a Master’s student in sociology with previous interview experience. All interviewers were trained by the first and last author, two psychologists (K.P. and S.M.-P.).

A semi-structured interview guide (available in Appendix A) was developed as a means of support for the interviewers. The guide was developed based on theory (e.g., theory of division of responsibilities, [3]) and previous studies (e.g., [7]), but tailored to this study’s specific objectives and adapted to the French food culture. In France, it is, for example, common to consume a multiple-component lunch and dinner (starter, main dish, dairy product, dessert), and this could have implications for portioning practices. For children in France, the consumption of a mid-afternoon snack (“goûter”) is also a common practice; it is even seen by many as an additional meal [25] (pp. 1–4).

The interviews always started with a short introduction of the interviewer and a brief review of the study information and data protection policy. Parents were asked to verbally confirm that they still agreed that an audio recording could be made and were asked to avoid the use of personal names, if possible. If parents had more than one child aged between 3 and 5 years, the interviewer specified which child would be the focus for the interview, chosen based on the child’s age or birth order. The interview guide comprised four main topic sections: (1) meal organization and composition; (2) meal service and portion sizes; (3) family rules around eating; and (4) the child’s appetite, satiation, and weight. For each section, there were a number of core questions, optional questions, and probes. To obtain realistic descriptions, the interviewer tried to limit the questions to a description of the meals and practices of the previous day, unless parents indicated that this day was very different from the family’s usual eating habits. In addition, a number of questions did not focus on the previous day, but aimed to obtain a more general description of practices or eating behaviors (e.g., “*How would you describe your child’s appetite*?”). At the end of the interview, parents were asked if the COVID-19 pandemic (still) had an impact on their current eating and feeding habits and thus if their descriptions during the interview differed from their usual habits. They were also invited to share additional information that had not yet been addressed and that they considered important. When they had nothing further to add, they were thanked for their time and the following steps of this study were explained to them.

The interview guide was pretested by inviting four parents (one father and three mothers) for a telephone interview. The interviews all went well and only minor adjustments to the guide were made, such as changing the order of certain questions and adding some notes addressed to the interviewers. The data of these four parents were therefore also included in this study’s analyses.

#### 2.3.3. Final Survey

After the interview, parents received a web link to a final online survey with questions complementing the data of the recruitment form and the interview. Parents were asked to complete information about the child (birth date, sex, weight, height) and about themselves (weight, height, work status, financial status). Due to the COVID-19 situation at the moment of data collection, children’s and parents’ weight and height were parent-reported; for the child, they were retrieved from the child’s health book or measured by the parent himself/herself. Parents were also asked to answer questions regarding the following topics.

*Self-regulation of the child.* To estimate how parents rate their child’s capacity to decide appropriate portion sizes for themself, they were asked to complete the following phrase: “If I did not guide the portion size of my child at mealtime…”. Parents could choose between: (1) “… (s)he would be able to choose an appropriate portion size”, (2) “…(s)he would serve too large portions”, or (3) “…(s)he would serve too little”. 

*Self-efficacy for identifying appropriate portion sizes.* One item was used to estimate how parents rate their own capacity to decide appropriate portion sizes for their child (“I am confident that I know appropriate portion sizes for my child’s meals”). They were asked to rate their answer on a 5-point scale ranging from “Do not agree at all” to “Totally agree”. This item was selected from the self-efficacy scale of Fulkerson et al. [26] and translated to French for this study.

*Information sources.* Three questions were used to obtain insight into possible sources of information for parents regarding portioning practices. First, they were asked if they “looked or asked for advice for the determination of portion sizes” (no or yes + description if yes). Then, they were asked if they were “knowledgeable about recommendations regarding determining portion sizes for children” (no or yes + description if yes). Finally, they were asked if they were “interested in receiving recommendations or advice that could guide them in determining portion sizes for their child” (5-point scale ranging from “I’m not at all interested” to “I am very interested”). 

The survey also included items to study children’s eating behaviors (appetite, food enjoyment, food neophobia, and food pickiness), parents’ use of pressure to eat, their level of restrained eating, and their motivations when buying food for the child, but these results will not be presented in this paper.

### 2.4. Data Transcription and Data Analysis

Interview recordings were transcribed verbatim. A thematic analysis was conducted following the steps of Braun and Clarke [27]: (1) familiarization with the data, (2) initial coding generation, (3) searching for themes based on initial coding, (4) review of the themes, (5) theme definition and labelling, and (6) report writing. 

The familiarization step (1) took place both throughout the data collection phase (listening to recorded interviews) and after (reading the transcribed interviews). This aided us in identifying preliminary patterns in the data and determining when the “saturation” of the data occurred, meaning that new interviews did not provide additional information to the data of the previous interviews. The first author had regular discussions with the interviewers about the interviews and the emerging patterns in the data. After the data familiarization phase, a group meeting (K.P. + S.M.-P. + V.F. + A.R.) took place to discuss the emerging themes. Then, three interviews were selected for independent initial coding by all researchers who were present at the group meeting. A data-led approach was used, but coding was also partly guided by the topic sections of the interview guide. After the independent coding phase, which was conducted by three researchers (A.R. + V.F. + K.P.), the initial codes and associated themes and subthemes were discussed in the group until a consensus was reached, resulting in a coding template. This template was used for the subsequent coding of the interviews. When doubts or difficulties arose among the coders regarding the placement of quotes, new group discussions took place and the coding template for themes and subthemes was revised where needed until agreement was reached between the coders. When all the interviews were coded, K.P. and S.M.-P. reviewed, regrouped, and defined the themes and subthemes that were of particular interest for answering the research questions of the current study and discussed the results of this process with S.I.

### 2.5. Trustworthiness and Translation of Quotes

Member checking, a respondent validation technique, was applied in order to confirm the interpretation of the data and increase the trustworthiness of the data [28]. After each interview, a summary of the interview was written by the interviewer and discussed with the first author. The summary was sent to the participant for validation and participants were invited to share additional information that had come to mind after the interview [29]. In some cases, the research team added a specific question for the participant in order to obtain additional information about a certain topic or to make sure that they fully understood certain statements made by the participant.

A number of quotes were selected for this article to exemplify the results of the thematic analyses. They were translated from French to English by a native English linguist who is fluent in French and has been living in France for many years. The original French quotes with their translation are presented in Appendix B. Brackets with dots in a quote (“[…]”) indicate that a number of words or sentences have been skipped.

### 2.6. Data Analysis of the Survey

Descriptive statistics were used to describe the participants’ characteristics and quantify the responses to the questions asked at the recruitment stage and in the final survey.

## 3. Results

A variety of themes and subthemes emerged from the analysis; an overview is presented in Table 2. Details about the themes and subthemes are described below.

### 3.1. Food Habits and Composition of Meals

Despite some minor deviations, all parents described that they follow the “French eating model” in their family: three meals a day (breakfast, lunch, dinner) and a mid-afternoon snack for the child. Meals are usually consumed at set times and at the table in the company of other family members.

A milk bottle, cereals with milk, and bread are common breakfast foods/drinks. Lunch and dinner usually consist of different components: a starter (salad), a main dish (proteins), cheese or yoghurt, and a dessert (fruit or a sweet dessert). For the mid-afternoon snack, food pleasure takes a central role in most families. Usually, something sweet is consumed, e.g., biscuits, cake, fruit compote, cream dessert, (drink) yoghurt. 

Food habits may alter between weekdays and weekend days, but in most families the difference is fairly limited. If changes occur, they mostly concern the timing of the meals or the extent of the meal—i.e., they may be more elaborate or festive on weekend days, especially when guests are invited.

### 3.2. Parental Portioning Practices

#### 3.2.1. Who Serves/Who Decides on Portion Sizes?

##### First Serve Versus Following Serves

In most families, a parent serves the pre-schooler and decides the portion size served. However, differences were found between the first serving and any subsequent servings. For the first serving, it is almost exclusively a parent who serves and decides. Most parents decide on a minimum portion the child should consume, then, if the child is still hungry or if he/she wants more, the child may receive or take another portion of the dish. At this point, the child usually has some say in how much is served, based on their expressions of hunger or their demands, or they may be allowed to serve themself.

*Indeed, I decide on the first serve. And then, if he’s still hungry, I serve again and I ask him how much he wants.*

*(U065)*

*Often, we serve him the first time. Then after, if they want seconds, I suggest they help themselves.*

*(TAL01)*

##### Different Practices for Different Meals?

Differences in serving practices were observed between different meals. For lunch and dinner, parents are mostly in control and little autonomy is granted to the child in terms of serving themself and for determining portion sizes, especially for the first servings (as described above). However, children are often allowed to take some cheese or a yoghurt and a dessert themselves, which are mostly products with a predetermined quantity.

*I’m the one who serves. Well, it’s me or my husband. We serve the children, yes.*

*(Y214)*

*Yes, like at lunchtime in fact, I serve up on plates and then dish them out to everyone.*

*(C697)*

*However, I let her have her yoghurt, for example at lunchtime they can choose which yoghurt they want. So she can go and open the fridge when I tell her she’s allowed to.*

*(P078)*

For breakfast, children participate more actively in serving themselves or in choosing what to eat (e.g., taking the food they want, pouring milk on their cereal), even though there were also some families where the parents prepare and serve breakfast for their child. According to some parents, what and how much the child eats at breakfast is strongly based on habits, which means that they have to exercise less control.

*At a push, the moment he guides me the most is in the morning about the quantity of milk or cereals or regarding toast, he tells me what he wants in terms of quantities but not for the other meals, I’m the one who decides.*

*(R863)*

*Well, in the morning, they get up and they can help themselves. Well, I check what they take, but as it’s always the same thing and the same portion size, let’s say I’m not surprised. They don’t take advantage in terms of what they take, what she takes.*

*(J086)*

For the mid-afternoon snack, there is also more child autonomy, especially in terms of food choice. Children can often decide what they want to eat and are allowed to take it themselves.

*Like in the morning, she chooses what she wants to eat, what she wants to drink. She helps herself to cakes in the cupboard, she’ll ask me for a drink but generally I give her water.*

*(S615)*

*After, it’s true that I leave them more often than not to choose their own snacks at home. I mean, I put things on the table and then I sort of leave them to it.*

*(TAL01)*

##### Conditioned Autonomy Child

As illustrated above, some parents grant more autonomy to their child than others. This ranges from no autonomy (not for serving, not for determining portion sizes), to interaction and discussion with the child about the portion size served by the parent, to allowing the child to serve himself/herself. However, even when the child is invited to say how much (s)he wants to eat or when serving, the parent will always monitor and re-adjust when deemed necessary. The child is never granted full autonomy.

*He helps himself, but I still keep an eye on him.*

*(U065)*

*After, for everything else, I know pretty well how she eats, so I adapt, I ask her how much she wants and sometimes her eyes are “bigger than her belly”, so I adapt by saying “eat this first and if you want more, you can, but I think it’s already fine like that”.*

*(N675)*

##### Why Does Parent or Child Serve?

When asked why it is the parent or child who serves, parents gave several reasons. The most common reason was “practicality”. Parents serve, for example, because it is faster, because the cook prepares the plates in the kitchen for everyone, to avoid danger (e.g., child getting burned by hot foods or by hot pots and pans), to avoid messy situations (e.g., when foods are too liquid), or because the tools used to serve are not adapted to the child’s size or motoric skills.

*But still, most of the time we serve […] yes, it’s more practical and quicker to be honest with you.*

*(TAL01)*

*Very often we serve her, we serve her because everything is the same … Always because of dexterity. It’s not easy to serve yourself from a dish […] however, at the moment as we’re eating quite a lot of raw vegetables, I let her serve herself. For example, when we eat radishes, she helps herself to the radishes.*

*(R371)*

*So I’d say it depends in fact. I push him to be a bit independent and to do things for himself. But then, if things are too hot, or too runny, or not easy to serve, he doesn’t help himself … but if it’s simple things, I don’t know. If he wants to serve himself from the salad bowl and take the salad tongs and serve himself, I don’t mind.*

*(T261)*

Here, it is interesting to note that some parents cite practical reasons to defend their habits, but also admit that they really just want to be in control.

*I say to him “you help yourself but as it’s really runny I’ll do it with you” so I can control it too.*

*(N675)*

Another reason why parents serve their child, is because they say that for their child *“it’s a game”*; the child does not take serving and portioning seriously. During the interview, parents were asked if they thought their child was capable of serving himself/herself or determining appropriate portion sizes. Most parents answered that they think their child would be able to serve the food, but not the right portion sizes or the right proportions (i.e., a balanced meal). They thought that children would serve too much of the foods they like. One father explained that he thought his daughter *“has not yet acquired the notion of quantity*”. Here, many parents admitted that their answer was a guess, that it is what they *“think”* would happen, because *“they haven’t really tested it”* or *“they would be surprised”*. In the final survey, parents were invited to answer a similar question. When presented with the statement “If I did not guide the portion size of my child at mealtime…”, 12 parents chose the answer “… (s)he would be able to choose an appropriate portion size”, 17 parents chose the answer “…(s)he would serve too large portions”, and 7 parents chose “…(s)he would serve too little”. There were no differences in answers when comparing different groups of parents, e.g., groups based on the child’s age (3/4/5 years old), the child’s birth order (first child or not), or the parent’s level of education (low/middle/high).

Furthermore, some children are allowed to serve themselves simply because they request it:

*When she asks, she serves herself, there’s no problem.*

*(E492)*

*No, it’s him who asks. He wants to do it on his own.*

*(TAL03)*

One mother also explained that she serves the children because it was done that way in her childhood (inter-generational transmission):

*I serve the eldest too. It’s true that my parents served us when we were kids … Well, my mother served us and it’s true that it’s … Yes, I tend to serve everyone.*

*(Y214)*

#### 3.2.2. Portion Sizes

##### Rules around Serving and Re-Serving

Many families have clear rules about whether or not children are allowed a second serving and what can be re-served, especially at lunch and dinner. As mentioned previously, for the first serving parents usually serve a small portion size which the child should finish, then the child can (be) re-serve(d) if they are still hungry. Parents prefer this approach because it contributes to avoiding food waste, and some parents also describe that a small first portion encourages the children to eat. If children are served too much food from the start, they are less likely to consume what is served.

*I don’t like throwing food away too much, so I generally give him a portion that I know he’ll be able to eat. But I’d rather he asks me for more rather than leave it. So I don’t serve too much.*

*(T261)*

*I prefer to serve less and that he eats everything and then at the worst I give him more if he wants it, rather than serving a lot. I’ve noticed that if you serve a big amount straight away there are times when he’ll look at his plate and then he’ll have two bites and … Yes, he will stop. However, if we give him smaller quantities, he will eat more easily. He’ll take them more easily.*

*(Y214)*

In contrast, one mother stated that she wants to teach her daughter to take the right quantity from the start at the first serving, as she does not want to create the habit of re-serving:

*But when she helps herself […] let’s say she doesn’t serve herself enough, sufficient quantities so that she’ll want more. And that’s something I don’t want to teach her: to serve herself again. That’s it. She helps herself once and that’s it.*

*(K122)*

However, in most families, re-serving is allowed but under certain conditions. For example, re-serving is not unlimited, and a second or subsequent serving will always be smaller than the previous one.

*Yes, it’s me who decides, and when it’s something she’s really liked, she asks me for seconds […] In general I give her a little less.*

*(S615)*

*So I ask him if he is really sure [to want more] because I don’t want him to waste food. And if he’s sure, I give him a small portion more. I’d rather give him a little bit and then serve him again a few times, than give him too much and then he doesn’t want it at all.*

*(T411)*

Moreover, there are not only rules regarding the size of any re-serving, but also about what can be re-served. Most parents allow their child to re-serve foods that they consider healthy, (e.g., vegetables, fruit), but not “unhealthy, sugary” foods. Some parents also described that they prefer to limit the re-servings of the main dish in favor of having a dairy product and a dessert afterwards, while other parents prefer more re-servings of the main dish and limiting or skipping the dairy product and/or dessert.

*Yes, no, not dessert. If it’s something really sweet, I won’t give him more. A Danette or something like that … because it’s a dessert, full stop. But if he wants more pasta salad or vegetables, there’s no hassle.*

*(S986)*

*If he asks me for a second ice cream, no, that’s out. If he asks me for another slice of cheese, I give him a slice of cheese. If he wants a piece of fruit, he’ll get one. It depends on the food. If I think it’s not bad for his health, I give him more. They are not overweight, so there you go … If they’re hungry, they’re hungry.*

*(Y023)*

*Sometimes when they want more, I tell them “yes, but there’s something else, you can eat a yoghurt, fruit”.*

*(N675)*

*We already try to make sure that they eat their main course well, because that’s the priority in terms of balancing their diet. And then we’ll say that cheese and dessert are “extras”. It’s, if really he’s still hungry, we try to balance it out so that he … so there you go, the main course is enough for him and after, the dessert is a little more for pleasure.*

*(B681)*

##### Tricks to Determine Portion Sizes

Parents described using certain “tricks” when determining the appropriate portion sizes for their child. The most commonly used trick was serving the child’s food on a small plate or bowl, which helps parents to serve smaller portions. One parent even described how every family member has a different size of tableware adapted to their age and size:

At home, it’s a bit like the three bears. *Laughter* There’s the big bowl, the medium bowl and the small bowl. *Laughter* because it’s true that they are 10, 6 and 4 years old so the portions are adapted according to their height. That’s how I’d put it. It’s true that the youngest one, I give him smaller portions. Even if this means I’ll serve him again, well I prefer …

*(TAL01)*

Other parents do not use different tableware for the child, but rather adjust the child’s portions in proportion to the portions of other family members—for example, smaller portions than for older siblings, or half the portion of the parent.

*I adapt his portion. Already, compared to his brother and sister, I give him a smaller portion.*

*(L691)*

*It’s about … what … I don’t know … I mean, half of my plate.*

*(Y023)*

Another trick used by parents to limit portions, is buying individual packages of certain foods and drinks, e.g., compote, fruit juice, biscuits, cereal, yoghurt.

*Yes, I buy a lot [of food] in individual portions for the children. Especially compotes, fruit juices, these are things that I buy in individual portions. Even cakes, I try to buy things in individual portions. If it’s cookies that are packed in twos, things like that. It’s easier to carry and easier to ration.*

*(T261)*

A very limited number of parents also determine portions sizes by counting the number of spoons:

*So it’s, let’s talk in tablespoons, it might be easier. I would put two tablespoons in. I prefer to put less than he eats in one go and then at the worst I’ll give him more if he wants it rather than serving a big amount.*

*(Y214)*

##### What Guides Parents When Determining Portion Sizes?

Only a few parents described that they adapt the child’s first portion or the timing of the meals according to the child’s physical activity that day, previous intakes during the day, expressions of hunger, or sickness or fatigue. Nevertheless, all parents explained that they know very well when their child is hungry and how they express this. Sensations of satiation are more difficult for parents to read and are often not expressed verbally, but rather through their behavior (e.g., stopping eating, pushing plate away). 

Some parents also described that children’s food preferences play a role in the portion size served. If the food served is a less well-liked, parents tend to give smaller portions of this food than foods that are well accepted or liked:

*He likes carbs a lot, but he has trouble with tabbouleh. So if there’s only tabbouleh and fresh, raw vegetables, I’m bound to use smaller quantities because I know he won’t like it so he’ll eat less. So that’s it, but otherwise it’s the same all the time.*

*(S616)*

When parents were explicitly asked how they know what “appropriate” portion sizes for their child are, most parents explained that the determination is made based on “their intuition” or “previous experience”, or that it is based “on the feeling” or “on sight”. Parents say that they know their child’s appetite and how much the child normally eats, so based on these habits they know how much to give, as expressed by this parent: “it is based on what he eats regularly” (TAL05). Others described that they learned to adapt the portion sizes to their child based on their observations—for example, they learned to give larger portions because their child always asked for more food after the first portion.

*As he gets older, we increase the quantities a bit more because we can see it, for example we’ll try to … We’ll give him a quantity as we used to and then we’ll see that in the end he’ll have several helpings and then we’ll say “well, maybe the quantity is too small”.*

*(Y214)*

Conversely, some parents know that portion sizes are good because the child does not ask for more food and is not hungry in between meals. Some parents also refer to their child’s health status: the child is “in good health”, “not overweight”, or “full of energy”. The child’s weight was not a preoccupation for the parents in this sample; to illustrate this, they referred to the child’s position in the “growth curves” and as “being in the norms”.

*I know that the quantities of what she eats are adapted to her metabolism and that she doesn’t take more than what she needs and not insufficient either, because she’s healthy.*

*((N675)*

Some parents also feel confident about their portioning practices because they already have experience with the older siblings of the pre-schooler—for example:

It’s through experience … yeah, it’s my third one eh. So, I think I did well with the others. *laughter* so no change.

*(L691)*

Parents’ explanations during the interview were also reflected in their answers to the following statement in the survey: “I am confident that I know appropriate portion sizes for my child’s meals”. Most parents (*n* = 26) indicated that they agreed, three parents totally agreed, six parents indicated they were neutral about this statement, and one parent did not totally agree.

The results of the survey also indicated that the majority of parents (*n* = 31) do not search or ask for information. Those who do either ask the doctor for advice (*n* = 3) or consult books or the internet (*n* = 3). In the next question, only seven parents expressed that they know about recommendations for determining portion sizes: they referred to the national health guidelines (PNNS) or knew about recommendations via their doctor, the internet, or an early childhood center. Finally, the majority of parents responded that they were interested (*n* = 16) or very interested (*n* = 10) in receiving recommendations or advice concerning determining portion sizes for their child. The other parents were either neutral (*n* = 6), not interested (*n* = 3), or not at all interested (*n* = 1). 

### 3.3. Family Rules around Eating

#### 3.3.1. Meal Timing

Most families eat at set times, especially during weekdays. When the child is hungry before mealtimes, parents usually explain that it is not time yet to eat and that they will have to wait. However, some exceptions exist. If it is very close to meal time, the child usually has to wait. If there is still a lot of time between the moment when the child expresses hunger and meal time, the timing of the meal is sometimes pushed forward or the child is given a little snack (e.g., bread, cheese, raisins):

*Well, yes … it all depends on the time. I explain to her that it’s soon [mealtime] or I’m still attentive obviously about when she eats. If she’s a bit hungry earlier, we can move her mealtime.*

*(TAL06)*

*It depends on the time. If it’s 10 min before the meal, no. If it’s an hour before, yes I give her a little something*

*(TAL07)*

#### 3.3.2. Pressure to Eat/Negotiating/Bribing

In addition to eating at set times, parents described a number of feeding practices that are used to make their child eat. As described previously, many parents serve their child a small first portion which the child should finish, then the child can (be) re-serve(d) if they are still hungry. Parents explained it is not always evident that the child will eat this first portion. If this is not the case, most parents will make an effort to encourage their child to eat a little more, and they will insist to a certain extent. Some will do so by saying how many spoons the child still needs to eat or by indicating the amount on the plate that they should finish. Others will negotiate with their child and some will bribe the child—for example, if the child does not eat the predetermined quantity there will be no dessert, or they cannot go for a walk together after the meal.

*Yes, when there’s a little bit left and I want him to eat more, I tell him “Well you eat two more spoons”. And then you leave the rest …*

*(TAL05)*

*In general, we try between brackets to blackmail him a bit, even if it doesn’t necessarily work. We tell him there’s only this to eat you see. We tell him there’s only this to eat and if he doesn’t eat, well there you go, there won’t be any cheese or dessert.*

*(B681)*

In contrast, a limited number of parents indicated that they avoid urging their child to eat more. They maybe only insist on making their child taste the food offered:

But there’s no such thing as “you finish your meal and you get your dessert”. But no, there isn’t. No *laugh* yes, no no there is none of that.

*(PRTAL03)*

*So I don’t actually force him to finish the plate and eat. […] In fact, except when I insist that he tastes something because I think it’s important to taste. Afterwards I don’t ask him to eat, I ask him to taste […] When you’re not hungry anymore, you’re not hungry anymore […] So I don’t force him to finish.*

*(T261)*

#### 3.3.3. Origins of Feeding Practices and Inter-Generational Transmission

According to several parents, their own or their partner’s education in childhood has influenced the establishment of their current feeding practices. They learned from their own parents that it is important to at least taste the foods served, and/or to finish the plate and to avoid waste. They also want to transfer these rules and values to their own children.

And then it was, yes we used to finish off our plates. My mother is like me, she doesn’t really like it if there are leftovers, so we prepare just about the right quantity for the meal for everyone, so … So yes, I think that’s my way of doing it too *laughter* getting it from my family, my parents

*(C697)*

In addition to passing on rules and values from their own childhood, some parents also explained what they want to teach their child about meal timing. As described above, several parents teach their child to be patient and to wait to eat until it is time to eat. Related to this, some teach that it is time to eat when it is mealtime or when everyone is sitting at the table, and that is not good to eat or snack later when the table has been cleared.

*Well, snacking … That, to be sure, is out of the question. When she tells us she’s hungry, we tell her, for example, “Well, you should have eaten earlier.”*

*(J086)*

Several parents also teach their child that they should only ask for food when they are (still) hungry and that they should only ask for what they are able to eat. Here, it is important that the child learns to listen to his/her stomach, or to eat in compliance with their sensations of hunger.

*I start from the principle that she has to work it out for herself—If she asks for something to eat, it’s that she wants it or she is hungry*

*(N675)*

*So for the quantity, we ask them at the end of the meal to listen to their bellies, if they want more or if it’s just greediness, so by hearing it time and again I think they have understood the difference.*

*(PRTAN01)*

As cited above, some parents also explained that they want their child to differentiate between wanting to eat because they are hungry or because they want to treat themselves (“gourmandise”). One parent also explained the importance of transferring pleasure in eating to the child:

*Well, she loves it, I’m not going to say no to her, no. […] I really want it to remain a pleasure to eat. Me, I love it, I love to eat, I love to cook and I really want it to be a pleasure for her.*

*(K122)*

## 4. Discussion

**Figure 1:**
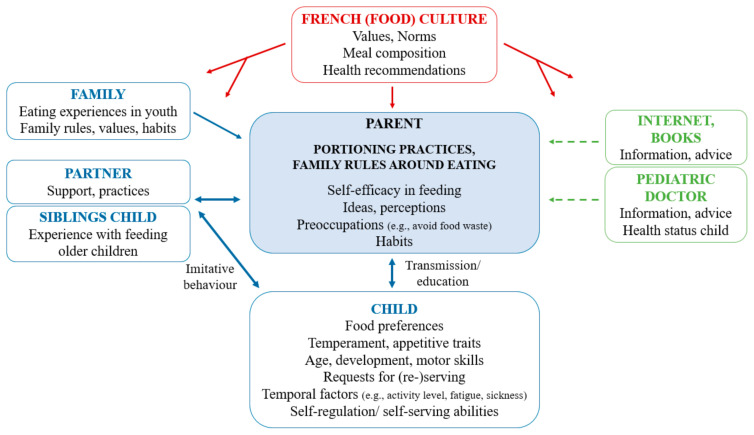
Schematic overview of parent-related factors, child-related factors and external factors influencing parental portioning practices and general feeding practices. Dotted lines indicate a minor influence.

The results of this study provide insight into the portioning practices and family rules around eating used by the parents of pre-schoolers in France, as well as into the drivers of these practices and rules. A schematic overview of the influencing factors identified in the present study is shown in Figure 1.

In accordance with the literature review of parental food and beverage portioning practices by Kairey et al. [16], parent-related factors, child-related factors, and external factors were all identified as influencing factors. This finding is in line with Bronfenbrenner’s ecological systems theory [30], which states that children develop in a complex system of relationships that are influenced by multiple levels of the environment, ranging from the child’s immediate home environment (microsystem) to their larger environment, encompassing culture, norms, and values (macrosystem). Following this theory, we can indeed assume that children’s capacity to self-regulate their food intake is influenced by factors on different levels, such as by people/factors in their microsystem, e.g., parents and their food portioning and feeding practices. In accordance with the theory, we observed that parents and their practices are in turn influenced by their child’s behavior and characteristics (bidirectional interactions between parent and child). Parents are also influenced by other people in the child’s microsystem (siblings, other family members, the pediatric doctor) and by factors in the macrosystem (culture, norms, health recommendations, etc.).

In this study, the division of responsibility between parent and pre-schooler in terms of determining portion sizes and serving was explored (Satter’s theory [3]). Overall, even though there was some variation in the degree of autonomy granted to children in this study, most pre-schoolers were granted little autonomy to serve themselves or to determine their own portion sizes. French parents are in control, or they at least guide the child or monitor what (s)he is doing. This is similar to the findings of a recent study of Loth et al. in the USA [7]. From the interviews, it is clear that French parents (try to) balance different elements: they want to be responsive to children’s expressions of hunger and to take their food preferences for portioning into account (child-related factors), but at the same time they also want their child to eat a minimum portion, to taste the foods offered, and to avoid food waste (parent-related factors).

In theory, young children are believed to be able to self-regulate their energy intake [4]. Following this idea, Satter [3] suggested that children should decide how much they eat and if they want to eat. In practice, this does not seem to be entirely true: studies with infants and pre-schoolers have shown, for example, that they do not adapt their energy-intake if they were offered a snack before a meal [31,32]. In addition, a lot of inter-individual variability exists with regard to children’s self-regulation capacities [33,34,35] and with regard to children’s eating responses to portion sizes [36]. It may, therefore, not be advisable to give children complete autonomy over their intake (how much). Instead, it is important that parents provide adequate structure and guidance for their child and that they are responsive to their needs. Most parents in this study reported recognizing their child’s hunger signals and “knowing” their child’s appetite. They prefer to start with a small portion of food and then re-serve foods based on the child’s demands. Moreover, they avoid serving portions that are too large and they do not insist that the child finishes the entire plate. Yet, only a handful of parents (*n* = 5) described that they explicitly teach their child to listen carefully to their stomach and their sensations of hunger when serving or asking for food or to stop when they are full. This awareness of the relationship between food and sensations of hunger should be encouraged among parents and children. This could prevent children from learning to eat for external reasons, such as because food is available or to bring comfort. This approach could also encourage parents to listen more carefully to children’s sensations and to explore, together with the child, how (s)he can obtain more experience and more autonomy in determining appropriate portion sizes for himself/herself. The results of this study show that parents have little confidence in their children’s self-regulation and self-serving capacities, often because parents have never explored this with their child.

In line with the results of previous studies [7,37], more autonomy was given to the child when serving breakfast or particularly when serving the mid-afternoon snack than when serving other meals. These studies, conducted in the USA, showed that often more flexibility is given with regard to the choice of foods eaten when snacking and when and where the child snacks. In response, Loth et al. [7] discussed that it is desirable for parents to provide sufficient structure, as snacks often make up a large share of the number of daily calories consumed by children [38]. In France, we also found that children were given more autonomy concerning choosing and serving snacks, but that an important difference from countries such as the USA lies in the structure around snacking. In many families in France, giving children a mid-afternoon snack (“goûter”) is a common practice: 62% of children aged 1–17 years consume it daily [39], and it is often considered as an additional meal for children. Families usually have rules with regard to what, when (often between 4.30 and 5.30 p.m. [40]) and where to snack, and there is little additional snacking throughout the rest of the day. Moreover, it is interesting to know that the mid-afternoon snack is a recommended practice by The French National Nutrition and Health Program (PNNS) to diversify children’s diet and ensure that they have energy throughout the day [25]. However, this does not mean that the mid-afternoon snack in France is nutritionally adequate [39]. For many, this eating occasion is a moment of pleasure and sweet foods are usually consumed [41]. Despite the adequate structure surrounding the mid-afternoon snack in France, it is therefore still advisable to encourage parents to limit children’s choices of snacks that are high in energy and encourage the consumption of a range of healthier snacks, without sacrificing the pleasure that comes with this eating moment [42].

In addition to the tradition of the mid-afternoon snack for children, it was clear during the interviews that the French food culture, passed on over generations, plays an important role in parental practices. Despite some minor deviations, all parents described that they follow the “French eating model” in their family [43]: three meals a day (breakfast, lunch, dinner) and a mid-afternoon snack for the child. Meals are usually consumed at set times and at the table in the company of other family members. In most families, lunch and dinner also consist of different components (i.e., starter, main course, dairy product, dessert), and parents strive to offer a variety of foods to their family, in accordance with the French health recommendations that stimulate the intake of a varied and balanced diet [44]. This preoccupation also has a clear impact on parental portioning practices: many parents want their child to at least taste all the foods offered, and they decide whether the child is allowed to have an extra helping depending on the types of foods (s)he wants. If the child still wants some “healthy” foods, (s)he is most likely allowed, but if (s)he wants more dessert it will be refused. It must be noted, however, that these portioning strategies based on the types of foods consumed have also been described by parents in other countries (e.g., [45,46]; additionally, see the review by Kairey et al. [16]). Nevertheless, it is possible that when striving to achieve a diverse diet, the use of different meal components in France may cause parents to automatically limit the size of children’s food portions. This is necessary in order to allow the child to eat a variety of foods at mealtime. In anticipation of the following components, it could be assumed that children may also subconsciously learn that they should not overeat right away but rather eat in moderation, otherwise they will not be able to eat all components of the meal, and in particular, will not be able to enjoy the dessert (which is probably their favorite dish).

The conscious focus of parents on food diversity stands in contrast with the rather unconscious, intuitive actions used for portioning foods for children. Parents know very well which foods they want their child to eat, but determine the portion given based on “their intuition”, “previous experience”, “on the feeling”, “on sight”, or on their child’s food preferences. This has also been described by parents in other qualitative studies (see review by Kairey et al. [16]). However, in some of these previous studies, parents have expressed doubts and difficulties with regard to determining appropriate portion sizes for their child, while the parents in the present study expressed that they feel confident in these intuitive practices and do not know of or look for recommendations. In France, recommendations about portioning practices are very limited. The French high council of public health [47] has formulated some recommendations regarding portion sizes for children aged 0–3 years and 3–17 years. They stressed the necessity of adapting portion sizes to the child’s needs and that there is no need to worry if the child follows a homogeneous growth trajectory (pp. 29–30). It is indeed very important that portions are adapted to children’s individual characteristics. Furthermore, it is interesting to note that the council did not give specific quantitative indications about appropriate portion sizes, but only comparative indications—for example, for children aged between 3 and 6 years, they state that the recommended portion size corresponds to about half the portion size of an adult (pp. 29–30). This comparative indication may be misleading and is obviously only advisable if parents eat an appropriate portion size. Johnson et al. [48] found that the amounts parents served themselves are indeed significantly positively associated with the amounts they served their pre-schoolers. Some caution is thus needed when communicating recommendations that use parental portions as a reference. Since parents’ portioning practices are rather unconscious and based on habits, it could be an interesting strategy to use nudges or to make changes in families’ environments in order to influence children’s intake in an unconscious way [49]. Both Robinson and Matheson [49] and Hetherington and Blundell-Birtill [50] identified the use of downsizing strategies—for example, using smaller tableware or purchasing individual small packages of food—as particularly interesting for supporting parents in serving appropriate portion sizes to children (and adolescents). Some parents in the present study also described using these strategies and finding them helpful for determining appropriate portion sizes for their child (to avoid giving portions that are too large). It is, however, uncertain as to whether parents who do not use these strategies currently will be receptive to recommendations about them. Parents in this study expressed some interest in receiving recommendations or advice concerning determining portion sizes for their child only when completing the final survey after the interview, but it is possible that parents showed this interest merely to please the researchers (social desirability). Population-based research is needed in order to assess this parental interest more properly. In any case, since parents appear to be quite confident in their portioning practices and do not actively search for information, it could be challenging to find a medium to convey recommendations or advice. 

In addition, it is important to point out that not only parents but also the government and food industry can play an important role in stimulating the intake of appropriate portion sizes. It is necessary, for example, to make sure that portion sizes of products do not increase for reasons of industrial benefit and that the portion sizes given to children are generally not too large. To illustrate, cultural differences exist in this matter, e.g., in France, portion sizes are generally smaller in restaurants, supermarkets, and cookbooks compared to in the USA [51].

### Limitations and Strengths

A limitation of this study may be the lower number of parents of 5-year-old children (*n* = 8) included compared to parents of 3-year-olds (*n* = 17) and 4-year-olds (*n* = 12). Even though we did not observe any noticeable differences according to the child’s age during the analyses, this may simply be due to the observed imbalance in age groups. For some parents, the child’s age, his developmental stage, or motoric skills were factors that influenced their decision to grant or not grant some autonomy to their child. When interpreting the results, it may therefore be important to keep in mind that they mostly reflect the practices and perceptions of parents with younger pre-schoolers. It would therefore be interesting to conduct studies with parents of older children using more balanced samples in order to study if parental portioning practices and the division of responsibility change with the child’s age and developmental skills, as well as which (and how) factors contribute to these changes. In addition, most children in this sample were at a healthy weight. Parental portioning practices could differ depending on the weight status of the child, and this should also be examined in future studies. Finally, relating to these points, despite efforts to recruit a large and diverse sample of parents for this study, which is definitely also a strength of this study, it is possible that the parents who volunteered to participate had an above-average interest in their child’s or family’s eating behavior. The described practices and influencing factors should therefore not automatically be generalized to all parents in France. The number of fathers participating was also quite limited, meaning that the views of mothers may be overrepresented in this study. The results of this study are also based on what parents declared; thus, an observational approach (i.e., observing practices in the home environment and the actual portion sizes served to the child or using digital food photography for observation) could be a valuable contribution to the field. 

Nevertheless, the interviews with a large and diverse sample of participants provided interesting insight into parents’ portioning practices for pre-schoolers within the context of the French (food) culture, a matter that is relatively still unexplored. The combination of interviews and short surveys enabled us to obtain deep insight and explanations, as well as allowing us to quantify certain perceptions, practices, and interests. The results of this comprehensive study are therefore valuable, as they provide, at the same time, insight into (1) parental portioning practices for different meals; (2) the division of responsibility between parent and child in terms of determining portion sizes and serving; (3) the factors influencing parental practices on different levels; and (4) parents’ confidence about their practices, the sources of information they use, and their wish for guidance. To our knowledge, previous studies undertaken in other countries have focused only on some of these components and never them in combination. 

Similarities in practices and influencing factors were observed between this study in France and studies conducted in other countries (mostly the USA). For instance, it seems generally to be difficult for parents—whatever their culture—to give autonomy to their pre-schooler and to let him/her to decide how much to eat, except in very specific meal contexts (e.g., at breakfast and when snacking). However, some cultural specificities have also been described that are likely to have a more subtle impact. For example, in many French families, lunch and dinner consist of different successive components (i.e., starter, main course, dairy product, dessert), which may cause parents to automatically limit the size of the portions of each of these components in order to stimulate a diverse eating pattern in the child and maintain their motivation to eat all the different components. In France, portion sizes are generally also smaller in restaurants, supermarkets, and cookbooks than those in the USA [51]. These cultural norms can also influence what parents think appropriate portion sizes for their child are, although this topic was not specifically examined in the current study. A quantitative study comparing actual given portion sizes could help us to clarify this issue. In addition, it was interesting to observe that French parents were generally confident about their food portioning practices, while some parents in previous studies have expressed limited knowledge regarding determining appropriate portion sizes for children and themselves. 

In short, the results of this study in France expand the results of previous studies in other countries and provide valuable insight for the possible guidance of parents and health campaigns in France and beyond.

## 5. Conclusions and Implications

This study revealed how French parents determine portion sizes for their pre-schoolers and how this responsibility is divided between both parents. Influencing factors related to the parent, child, and social environment were identified, as well as specificities related to the French (food) culture. Most parents are in control when serving and portioning food, but, at the same time, they are also responsive to the child’s requests and characteristics. For parents, portioning food is an intuitive action that is guided by habits, their experience, and “knowing their child”. They are confident about their portioning skills and most of them declare that they do not search for information to guide them in this action. Nevertheless, even though parents seem to adopt responsive portioning practices, it may be important to encourage them to be more aware of their children’s capacity to self-regulate their food intake and how to stimulate this capacity. Parents can, for example, help their children to listen to their inner sensations of hunger and fullness and encourage them to adjust their intake to match this. Parents can grant their children some autonomy/responsibility in this action, adapted to the child’s age and development. Downsizing strategies could also be recommended to parents. Since parents will not look for this guidance, it may, however, be challenging to find a way to pass on these recommendations. Industries and governments should also be encouraged to take responsibility and limit the portion sizes of products made for children.

## Figures and Tables

**Table 1:** Characteristics of participating parents (*n* = 37) and their children.

Parents’ Characteristics:	*n*
Sex (female/male)	32/5
Age in years, mean (min.–max.)	33.9 (23–42)
Relationship status (couple/single parent)	30/7
Weight status:	
Underweight (BMI < 18.5)	4
Healthy weight (18.5 ≤ BMI < 25)	18
Overweight (25 ≤ BMI < 30)	9
Obesity (BMI ≥ 30)	4
Unknown	2
Level of education:	
Low (no diploma, high-school diploma, higher technology degree)	11
Middle (two or three-year higher education degree)	16
High (Master’s degree or higher)	10
Work status:	
Working (part-time or full-time)	28
Unemployed, job seeker	3
Student	1
Other (e.g., parental leave, parent at home)	3
Unknown	2
Perception of financial situation:	
You can’t make ends meet without going into debt	1
You get by but only just	0
Should be careful	8
It’s OK	22
At ease	3
Unknown/Does not wish to answer	3
**Children’s characteristics:**	
Age:	
3 years	17
4 years	12
5 years	8
Birth order:	
Firstborn or only child	20
Child with older sibling(s)	17
Weight status ^a^:	
Underweight	5
Healthy weight	27
Overweight	3
Obesity	0
Unknown	2

^a^ Weight categories for children corresponding to BMI-for-age percentiles based on growth charts for children and teens ages 2 through 19 years of the Centers for Disease Control and Prevention (CDC).

**Table 2:** Themes and subthemes resulting from the analysis.

Themes	Subthemes		
1. Food habits and composition of meals	1.1 French food culture	1.1.1 Different components meal	
		1.1.2 Nutritional values	
2. Parental portioning practices	2.1 Who serves/who decides on portion sizes?	2.1.1 First serve versus following serves	
		2.1.2 Different practices for different meals?	- Breakfast - Lunch - Mid-afternoon snack - Dinner - Milk bottle
		2.1.3 Conditioned autonomy child	
		2.1.4 Why does parent or child serve?	- Practical reasons - For the child, “it’s a game” - Self-regulation capacity child - Child’s demands - Influence other family members
	2.2 Portion sizes	2.2.1 Rules around serving and re-serving	- Quantity of food - Healthy vs. unhealthy foods
		2.2.2 Tricks to determine portion sizes	
		2.2.3 What guides parents when determining portion sizes?	- Child’s physical activity/intake previous meal/expression of hunger - Child’s food preferences - Parents’ confidence in own portioning practices - Information sources
3. Family rules around eating	3.1 Meal timing		
	3.2 Pressure to eat/negotiating/bribing		
	3.3. Origins of feeding practices and inter-generational transmission	3.3.1 Own experiences in childhood	
		3.3.2 Educational goals	

## Appendix A. Interview Guide to Semi-Structured Interview

(1) Researcher Introduction + study/consent form/questions

*Hello, my name is …I work at Dijon’s Centre des Sciences du Goût et de l’Alimentation. First of all, I wish to thank you for accepting to participate in this study. We are conducting interviews to gain greater knowledge on food practices in households with children aged between 3 and 5 years old. I propose that we talk about your child’s food habits. I wish to inform you that that anything you say will remain an anonymous contribution. If you agree, I will record this discussion to make sure that none of your answers will be misconstrued. Do you agree to this, are you ready to begin?*

(For the interviewer: remember to not pronounce the participant’s and child’s name once the recording has begun.)

**Table A1:** Containing the themes, questions and probes addressed during the semi-structured interview.

Theme	Questions	Probes
**Meal organization and composition**	*You have agreed to participate in this study because you have a young boy/girl who is …years old (state which child is being talked about)* *In this interview, we are going to focus on your …year old child’s eating habits.* *To begin this interview, could you describe what your child ate and drank yesterday between getting up and going to bed?* *Do you think that the day is representative of what he/she normally eats?* (If the response is no, describe the day before last) (don’t ask the question too quickly in the interview)	Indicate that this also includes any food outside of normal mealtimes (snacks…)
	Ask again about: snacks, drinks, anything eaten between meals. Explain clearly what is considered a snack. Indicate that a sweet or cake eaten on it own counts.	*If the child is hungry between meals, do you give a little something to eat to get him/her to wait?*
	*Do you maintain the same routine at the weekend?* (*‘the week’* if the interview is on Monday)? *What’s different?*	If the child has lunch with his/her nanny during the week, ask about food habits during lunch with the parents at the weekend.
**Meal service and portion sizes (for each meal)**	- *Thank you for this very precise description. We are now going to go into more detail about how each of these moments when food is eaten. To do so, we are going to look at each meal and each snack, and we are going to see how they happen…you will see it’s very simple.* - Is the food and are the drinks what are normally eaten during the meal?	
	*In what sort of conditions were yesterday’s meals taken?*	- *Where did the meal take place?* - *Who was with the child?* - *Were the people present also eating at the same time?* - *Was the food on the table to be served, or was it served away from the table, in the kitchen?* - *Where there any TV, Telephone, computer screens present too?* - *What type of dish did the child eat from? Was it different to that of the adult, other child?*
	*How do things take place when serving your child’s meal, when at home?* *Did your child serve himself/herself or was he/she served by someone else?* *How are the quantities fixed/chosen that are given to your child during the different meals of the day, during snacks?*	If someone served your child: - *Who served him/her? Why?* - *How was the portion size determined (thoughts/ideas?)* - *Did your child participate in determining the portion-size?* - Variation according to different situations? - Help for seasoning/sauces?
		If the child helped himself/herself: - *Did he/she help himself/herself on his/her own or did he/she receive a little help or instructions?* - *At what age did the child start serving himself/herself?*
	*Did he/she finish everything up?* Everything eaten on his/her plate at each meal? Ask for each meal	- *Otherwise: what happened?* - *If yes, did he/she ask for more? What did you do or say?*
	*If your child has finished everything and asks for more, how do you generally respond?*	*Do you give more? Do you move on to next food/course? Do you explain that the meal is over? Do you propose something else? Is it the same for each food that makes up the meal? And for each meal? Why (not)?*
	*And what do you do if your child refuses to eat something you’ve served, or a portion of something?*	
	*Are you satisfied with the amount of food that your child eats?*	
	*Do you think your child is able to serve himself/herself and fix the portion-size adapted to his/her needs?*	
	*How do you go about finding out what is the adapted portion-size for your child?* *More generally, what influences your food practices/habits (intuition, personal experience, family or friends, doctor, internet sites, blog…)? ….*	E.g., medical advice, school, child-care center, family or friends, internet, blogs, advertisements, books, reports… In the case of families with several children: *Has the way in which you handle food with your children changed? If yes, in which ways?* *Different approaches for different children?*
**Family rules around eating**	*Let’s now move on to look at rules or principles relating to food which are important for you. Very simply, in your household, are there rules relating to types of food you serve, for different meals…etc.…* (It’s very important to give the participant the necessary time to express spontaneously what is important for him/her, reword the question before trying to elicit again)	*(e.g., the child must try tasting everything, he/she only takes what he/she wants from the plate.)* - *Can these rules vary? How and why?* - *Does he/she have an unlimited access to certain foods? (Sweets …)* *What inspires you in the setting up of these rules? What motivates you to set up these rules?*
	*Would you say that your behavior/approach towards food is different to that of your husband/wife/partner)? Do you have the same approach or habits relating to food as your husband/wife/partner)?*	*Who fixes portion size?* *Differences between husband/wife/partner?* *Food choice?* *Rules relating to food?*
**Appetite, satiation and weight**	*We are now going to come back to your child, in particular about his/her appetite. How would you describe your child’s appetite? Would you say that your child’s appetite is stable, constant (has there been an evolution/change in it)?*	- *How do you know that your child is hungry? Does your child let you know when he/she is hungry?* - *How do you manage the situation when your child says he/she’s hungry/not hungry?* - *How do you manage things when your child tells you that he/she is still hungry for example just after the meal?*
	*Does your child let you know that he/she’s had enough to eat/is no longer hungry?*	- *Your child tells you that he/she’s had enough to eat/is no longer hungry and he/she has finished eating what’s on his/her plate, what do you do?* - *Your child lets you know that he/she has had enough, isn’t hungry anymore after the meal, but wants a dessert, how do you respond?*
	*We are now going to talk about your child’s weight and height.* *How do you manage this information? Do you make a note of the weight/height of your child? How often? Is your child’s growth a cause of concern for you? How do you track your child’s growth? Using which tools (Health notebook, wall chart?)*	- *For what reasons?* Through worry …?
**The current lockdown context**	*We have gone through an unusual period with the lockdown and the gradual lifting of it. So, we’d like to know if this period of time has modified your behavior and habits as you have described them during this interview.* *According to you, have there been any changes/an evolution in what you have just explained to me concerning the period before the lockdown?*	
**Additional participant comments**	*Thank you so much for answering my questions. Maybe you would like to talk about things which are important to you and that I’ve not asked you about?* *Are there any aspects that I’ve not mentioned and that you would like to add?*	

Interview conclusion + thanks and compensation + explanation of what follows

- *If you have nothing further to add, I wish to really thank for your time and for answering my questions.*
- *We would really appreciate it if you could complete a final questionnaire, online, which will take about 10 min of your time. You can fill-in this questionnaire at your convenience (give the person’s code). Once we have acknowledged receipt of this questionnaire, and to thank you for your time spent taking part in our study, we will send you a voucher of 20€ through the post.*
- *Do you have any final questions about this questionnaire or the study in general?*
- (Do not record) Check that the postal address is correct!

## Appendix B. Original French Citations and Their English Translation

**Table A2:** Containing the original French citations used as illustrations in the article and their English translation.

Original French citation	Translated English citation
**3.2 Parent portioning practices**	
**3.2.1 Who serves/ who decides on portion sizes?**	
**First serve versus following serves**	
*Effectivement au premier service c’est moi qui décide. Et puis ensuite, s’il a encore faim, je fais un deuxième service où là, je lui demande à quel point il veut être resservi. (U065)*	*Indeed, I decide on the first serve. And then, if he’s still hungry, I serve again and I ask him how much he wants. (U065)*
*Souvent on le sert nous la première fois. Mais après si c’est pour se resservir, je les invite eux à se resservir. (TAL01)*	*Often, we serve him the first time. Then after, if they want seconds, I suggest they help themselves. (TAL01)*
**Different practices for different meals?**	
*C’est moi qui sers. Enfin, c’est moi ou c’est mon mari. C’est nous qui servons les enfants à table, oui. (Y214)*	*I’m the one who serves. Well, it’s me or my husband. We serve the children, yes. (Y214)*
*Oui, comme le midi en fait, je prépare les assiettes et je les amène à tout le monde. (C697)*	*Yes, like at lunchtime in fact, I serve up on plates and then dish them out to everyone. (C697)*
*Par contre, je la laisse prendre son yaourt, par exemple le midi ils ont le droit de choisir le yaourt qu’ils veulent. Donc elle a le droit d’aller dans le frigo quand je lui donne l’autorisation. (P078)*	*However, I let her have her yoghurt, for example at lunchtime they can choose which yoghurt they want. So she can go and open the fridge when I tell her she’s allowed to. (P078)*
*A la limite, le moment où il me guide le plus c’est le matin sur la quantité de lait ou de céréales ou sur les tartines, il va me dire ce qu’il veut en termes de quantités mais sur les autres repas non, c’est moi qui impose. (R863)*	*At a push, the moment he guides me the most is in the morning about the quantity of milk or cereals or regarding toast, he tells me what he wants in terms of quantities but not for the other meals, I’m the one who decides. (R863)*
*Bon le matin, ils se lèvent et puis ils peuvent se servir eux-mêmes. Bon, je contrôle ce qu’ils prennent, mais bon comme c’est tout le temps la même chose en portion identique, je ne suis pas étonnée on va dire. Ils ne font pas d’abus sur ce qu’ils prennent, sur ce qu’elle prend. (J086)*	*Well, in the morning, they get up and they can help themselves. Well, I check what they take, but as it’s always the same thing and the same portion size, let’s say I’m not surprised. They don’t take advantage in terms of what they take, what she takes. (J086)*
*Comme le matin c’est elle qui va choisir ce qu’elle veut manger, ce qu’elle veut boire. Elle se sert dans le placard pour les gâteaux, elle va me demander pour boire mais en général je lui donne de l’eau. (S615)*	*Like in the morning, she chooses what she wants to eat, what she wants to drink. She helps herself to cakes in the cupboard, she’ll ask me for a drink but generally I give her water. (S615)*
*Après, c’est vrai que le goûter à la maison, je les laisse plus souvent en autonomie. Enfin, je mets des choses sur la table et puis, je les laisse un peu se gérer quoi. (TAL01)*	*After, it’s true that I leave them more often than not to choose their own snacks at home. I mean, I put things on the table and then I sort of leave them to it. (TAL01)*
**Conditioned autonomy child**	
*Il se sert tout seul, mais je surveille quand même. (U065)*	*He helps himself, but I still keep an eye on him. (U065)*
*Après pour tout le reste je connais un peu son appétit donc j’adapte, je demande déjà quelle quantité elle veut et des fois elle a « les yeux plus gros que le ventre » donc je réadapte moi-même en disant « tu manges déjà ça et si tu en reveux j’en remets mais déjà je crois que c’est bien comme ça ». (N675)*	*After, for everything else, I know pretty well how she eats, so I adapt, I ask her how much she wants and sometimes her eyes are "bigger than her belly", so I adapt by saying "eat this first and if you want more, you can, but I think it’s already fine like that". (N675)*
**Why does parent or child serve?**	
*Mais quand même la plupart du temps c’est nous qui servons […] oui, le côté pratique et rapide pour être très honnête. (TAL01)*	*But still, most of the time we serve […] yes, it’s more practical and quicker to be honest with you. (TAL01)*
*Très souvent on la sert, on la sert parce que tout pareil… Toujours à cause de la motricité. Ce n’est pas évident de prendre dans un plat et de se servir […] Alors par contre en ce moment comme on mange pas mal de crudités, je la laisse se servir. Par exemple, quand on mange des radis, c’est elle qui se sert des radis. (R371)*	*Very often we serve her, we serve her because everything is the same… Always because of dexterity. It’s not easy to serve yourself from a dish […] however, at the moment as we’re eating quite a lot of raw vegetables, I let her serve herself. For example, when we eat radishes, she helps herself to the radishes. (R371)*
*Alors je vais dire que ça dépend en fait. Je le pousse à être un peu autonome et à faire des choses par lui-même. Mais après voilà, si c’est des choses qui sont trop chaudes, ou trop liquides, ou pas faciles à servir, il ne se sert pas tout seul… mais si c’est des choses simples, je ne sais pas. S’il veut se servir dans le saladier et prendre la pince à salade et se servir, ça ne me dérange pas. (T261)*	*So I’d say it depends in fact. I push him to be a bit independent and to do things for himself. But then, if things are too hot, or too runny, or not easy to serve, he doesn’t help himself… but if it’s simple things, I don’t know. If he wants to serve himself from the salad bowl and take the salad tongs and serve himself, I don’t mind. (T261)*
*Je lui dis « tu te sers mais comme ça coule vite je fais avec toi » pour pouvoir contrôler aussi. (N675)*	*I say to him "you help yourself but as it’s really runny I’ll do it with you" so I can control it too. (N675)*
*Quand elle le demande, c’est elle qui se sert, il n’y a pas de soucis. (E492)*	*When she asks, she serves herself, there’s no problem. (E492)*
*Non, c’est lui qui demande. Il veut faire tout seul. (TAL03)*	*No, it’s him who asks. He wants to do it on his own. (TAL03)*
*Je sers le grand aussi. C’est vrai que j’ai des parents qui nous ont servi petits… Enfin, j’ai ma mère qui nous servait et c’est vrai que c’est… Oui, j’ai une tendance à servir tout le monde, moi. (Y214)*	*I serve the eldest too. It’s true that my parents served us when we were kids… Well, my mother served us and it’s true that it’s… Yes, I tend to serve everyone. (Y214)*
**3.2.2 Portion sizes**	
**Rules around serving and re-serving**	
*Moi, je n’aime pas trop jeter donc je mets en général une portion que je sais qu’il est capable de manger. Mais je préfère qu’il m’en redemande plutôt qu’il en laisse. Donc, je ne charge pas trop les assiettes. (T261)*	*I don’t like throwing food away too much, so I generally give him a portion that I know he’ll be able to eat. But I’d rather he asks me for more rather than leave it. So I don’t serve too much. (T261)*
*Je préfère en mettre moins qu’il en mange une fois et après au pire, je lui en redonne s’il en veut, plutôt que de lui mettre une grosse quantité. Pour l’avoir remarqué, si on lui met la grosse quantité dès le départ, il y a des fois où il va regarder l’assiette et puis il va en manger deux bouchées et… … Oui, il va s’arrêter. Que si on lui met des plus petites quantités, il en mangera plus facilement. Il en prendra plus facilement. (Y214)*	*I prefer to serve less and that he eats everything and then at the worst I give him more if he wants it, rather than serving a lot. I’ve noticed that if you serve a big amount straight away there are times when he’ll look at his plate and then he’ll have two bites and… …Yes, he will stop. However, if we give him smaller quantities, he will eat more easily. He’ll take them more easily. (Y214)*
*Mais quand elle se sert […] disons qu’elle ne met pas les quantités, ces quantités suffisantes qui font que derrière elle va se resservir. Et ça, c’est un truc que je ne veux pas lui apprendre en plus: à se resservir. C’est voilà. Elle se sert une fois et voilà. (K122)*	*But when she helps herself […] let’s say she doesn’t serve herself enough, sufficient quantities so that she’ll want more. And that’s something I don’t want to teach her: to serve herself again. That’s it. She helps herself once and that’s it. (K122)*
*Oui, c’est moi qui détermine, et quand c’est quelque chose qu’elle a bien aimé, là elle me redemande une 2ème fois […] En général je lui mets un peu moins. (S615)*	*Yes, it’s me who decides, and when it’s something she’s really liked, she asks me for seconds […] In general I give her a little less. (S615)*
*Alors, je lui demande s’il est vraiment certain [de vouloir plus] parce que je ne veux pas qu’il gâche la nourriture. Et s’il est sûr, je lui en remets une petite portion. Je préfère lui en remettre un tout petit peu et de lui en remettre plusieurs fois, que de lui mettre une trop grosse quantité et là après finalement il n’en veut plus du tout. (T411)*	*So I ask him if he is really sure [to want more] because I don’t want him to waste food. And if he’s sure, I give him a small portion more. I’d rather give him a little bit and then serve him again a few times, than give him too much and then he doesn’t want it at all. (T411)*
*Oui non, pas le dessert. Si c’est un truc hyper sucré, je ne lui en redonnerai pas. Une Danette ou un truc comme ça… parce que c’est un dessert et point-barre. Mais s’il reveut de la salade de pâtes ou des légumes, il n’y a pas de problème. (S986)*	*Yes, no, not dessert. If it’s something really sweet, I won’t give him more. A Danette or something like that… because it’s a dessert, full stop. But if he wants more pasta salad or vegetables, there’s no hassle. (S986)*
*S’il me demande une deuxième glace, non, c’est mort. S’il me redemande un bout de fromage, je lui redonne un bout de fromage. S’il veut un fruit, il va avoir un fruit. Ça dépend des aliments en fait. Si j’estime que ce n’est pas mauvais pour sa santé, je lui en redonne. Ils ne sont pas en surpoids, alors voilà… S’ils ont faim, ils ont faim. (Y023)*	*If he asks me for a second ice cream, no, that’s out. If he asks me for another slice of cheese, I give him a slice of cheese. If he wants a piece of fruit, he’ll get one. It depends on the food. If I think it’s not bad for his health, I give him more. They are not overweight, so there you go… If they’re hungry, they’re hungry. (Y023)*
*Des fois quand ils ont envie de se resservir je leur dis « oui mais il y autre chose, tu peux manger un yaourt, fruit ». (N675)*	*Sometimes when they want more, I tell them "yes, but there’s something else, you can eat a yoghurt, fruit". (N675)*
*Nous on essaye déjà d’une manière générale qu’il mange bien son plat principal parce que justement c’est plus la priorité par rapport au fait d’équilibrer son alimentation. Et après on va dire que le fromage et dessert, c’est « du plus ». C’est, si vraiment il a encore faim, on essaye de doser, voilà pour qu’il… voilà, que le plat principal lui suffise et après, voilà le dessert c’est un petit plus plaisir. (B681)*	*We already try to make sure that they eat their main course well, because that’s the priority in terms of balancing their diet. And then we’ll say that cheese and dessert are "extras". It’s, if really he’s still hungry, we try to balance it out so that he… so there you go, the main course is enough for him and after, the dessert is a little more for pleasure. (B681)*
**Tricks to determine portion sizes**	
*Chez nous, c’est un peu les trois ours. *rire* c’est voilà, il y a le grand bol, le moyen bol et le petit bol. *rire* parce que c’est vrai que du coup ils ont 10, 6 et 4 ans donc les portions sont adaptées en fonction leur taille. On va dire. C’est vrai que du coup le petit dernier, je lui donne des portions plus petites. Quitte à le resservir, enfin je préfère… (TAL01)*	*At home, it’s a bit like the three bears. *Laughter* There’s the big bowl, the medium bowl and the small bowl. *Laughter* because it’s true that they are 10, 6 and 4 years old so the portions are adapted according to their height. That’s how I’d put it. It’s true that the youngest one, I give him smaller portions. Even if this means I’ll serve him again, well I prefer… (TAL01)*
*Je lui adapte sa portion. Déjà, par rapport à son frère et à sa sœur, je lui donne une portion plus petite. (L691)*	*I adapt his portion. Already, compared to his brother and sister, I give him a smaller portion. (L691)*
*C’est à peu près… quoi… Je ne sais pas… je veux dire, la moitié de mon assiette. (Y023)*	*It’s about… what… I don’t know… I mean, half of my plate. (Y023)*
*Oui, j’achète beaucoup [d’aliment] en individuel pour les enfants. Notamment les compotes, les jus de fruits, c’est des choses que j’achète en individuel. Même les gâteaux, j’essaye de prendre des choses en individuel. Si c’est des cookies qui sont emballés par deux, des choses comme ça. C’est plus facile à emmener et plus facile à rationner. (T261)*	*Yes, I buy a lot [of food] in individual portions for the children. Especially compotes, fruit juices, these are things that I buy in individual portions. Even cakes, I try to buy things in individual portions. If it’s cookies that are packed in twos, things like that. It’s easier to carry and easier to ration. (T261)*
*Alors c’est, on va parler en cuillères à soupe, ça va peut-être être plus facile. J’y mettrais deux cuillères à soupe. Je préfère en mettre moins qu’il en mange une fois et après au pire, je lui en redonne s’il en veut plutôt que de lui mettre une grosse quantité. (Y214)*	*So it’s, let’s talk in tablespoons, it might be easier. I would put two tablespoons in. I prefer to put less than he eats in one go and then at the worst I’ll give him more if he wants it rather than serving a big amount. (Y214)*
***What guides parents when determining portion sizes?***	
*Il aime énormément les féculents, mais il a du mal quand c’est du taboulé. Donc s’il y a que taboulé et crudités, je vais forcément mettre des plus petites quantités parce que je sais qu’il ne va pas trop aimer donc il va moins manger. Voilà, mais après sinon globalement ça reste tout le temps pareil. (S616)*	*He likes carbs a lot, but he has trouble with tabbouleh. So if there’s only tabbouleh and fresh, raw vegetables, I’m bound to use smaller quantities because I know he won’t like it so he’ll eat less. So that’s it, but otherwise it’s the same all the time. (S616)*
*En grandissant, on augmente tout petit peu plus les quantités parce qu’on le voit, par exemple on va essayer de … On va lui mettre une quantité comme on mettait avant et puis on va constater qu’au final, il va nous en reprendre plusieurs fois et là on va dire « bon ben là peut-être la quantité est peut-être trop juste ». (Y214)*	*As he gets older, we increase the quantities a bit more because we can see it, for example we’ll try to … We’ll give him a quantity as we used to and then we’ll see that in the end he’ll have several helpings and then we’ll say "well, maybe the quantity is too small". (Y214)*
*Je sais que les quantités de ce qu’elle mange sont adaptées à son métabolisme et qu’elle ne prend pas plus que ce qu’il lui faut et pas non plus insuffisamment parce que voilà, elle est en bonne santé. (N675)*	*I know that the quantities of what she eats are adapted to her metabolism and that she doesn’t take more than what she needs and not insufficient either, because she’s healthy. (N675)*
*C’est selon l’expérience… ouais c’est ma troisième hein. Donc je pense avoir bien fait avec les autres. *rire* donc on continue comme ça. (L691)*	*It’s through experience… yeah, it’s my third one eh. So, I think I did well with the others. *laughter* so no change. (L691)*
**3.3 Family rules around eating**	
**3.3.1 Meal timing**	
*Ben si… tout dépend de l’heure. Je lui explique que c’est bientôt [l’heure du repas] ou voilà je suis à son écoute quand même par rapport au repas. Si elle a faim un peu plutôt, on peut décaler l’heure de son repas. (TAL06)*	*Well, yes… it all depends on the time. I explain to her that it’s soon [mealtime] or I’m still attentive obviously about when she eats. If she’s a bit hungry earlier, we can move her mealtime. (TAL06)*
*Ça dépend l’heure. Si c’est à 10 minutes du repas, non. Si c’est une heure d’avant, oui je lui donne un petit quelque chose (TAL07)*	*It depends on the time. If it’s 10 minutes before the meal, no. If it’s an hour before, yes I give her a little something (TAL07)*
**3.3.2 Pressure to eat—negotiate—bribe**	
*Oui, quand il en reste un peu et que je voudrais qu’il mange encore, je lui dis « ben tu manges encore deux cuillères. ». Et après, tu laisses le reste … (TAL05)*	*Yes, when there’s a little bit left and I want him to eat more, I tell him “Well you eat two more spoons”. And then you leave the rest … (TAL05)*
*En général, on essaye un peu entre guillemets de l’avoir au chantage, même si ça ne marche pas forcément. On lui dit qu’il n’y a que ça à manger quoi. On lui dit il n’y a que ça à manger et s’il ne mange pas bah voilà, il n’y aura pas de fromage ou de dessert. (B681)*	*In general, we try between brackets to blackmail him a bit, even if it doesn’t necessarily work. We tell him there’s only this to eat you see. We tell him there’s only this to eat and if he doesn’t eat, well there you go, there won’t be any cheese or dessert. (B681)*
*Mais il n’y a pas ce côté du genre « tu finis ton plat et tu as ton dessert ». Mais non il n’y a pas. Aucun *rire* oui, non non il n’y a pas ça. (PRTAL03)*	*But there’s no such thing as "you finish your meal and you get your dessert". But no, there isn’t. No *laugh* yes, no no there is none of that. (PRTAL03)*
*Donc je ne l’oblige pas justement à finir l’assiette et à manger. […] En fait, hormis où j’insiste les fois pour qu’il goûte quelque chose parce que je trouve ça important de goûter. Après je ne lui demande pas à manger, je lui demande de goûter […] Quand on a plus faim, on a plus faim […] Donc je ne force pas finir. (T261)*	*So I don’t actually force him to finish the plate and eat. […] In fact, except when I insist that he tastes something because I think it’s important to taste. Afterwards I don’t ask him to eat, I ask him to taste […] When you’re not hungry anymore, you’re not hungry anymore […] So I don’t force him to finish. (T261)*
**3.3.3 Origins of feeding practices and inter-generational transmission**	
*Et puis c’était, oui on finissait nos assiettes. Ma mère elle est comme moi, elle n’aime pas trop qu’il y ait des restes donc on fait des quantités à peu près juste quoi pour le repas pour tout le monde donc… Donc oui je pense que c’est mon schéma à moi *rire* de ma famille, de mes parents (C697)*	*And then it was, yes we used to finish off our plates. My mother is like me, she doesn’t really like it if there are leftovers, so we prepare just about the right quantity for the meal for everyone, so… So yes, I think that’s my way of doing it too *laughter* getting it from my family, my parents (C697)*
*Ben, le grignotage… Ça, c’est sûr, c’est hors de question. Voilà, nous quand elle nous dit qu’elle a faim, on lui dit par exemple « ben tu avais qu’à mieux manger tout à l’heure. » (J086)*	*Well, snacking… That, to be sure, is out of the question. When she tells us she’s hungry, we tell her, for example, “Well, you should have eaten earlier. ” (J086)*
*Je pars du principe qu’il faut qu’elle se rende compte que si elle demande quelque chose à manger, c’est qu’elle en veut ou qu’elle a faim (N675)*	*I start from the principle that she has to work it out for herself - if she asks for something to eat, it’s that she wants it or she is hungry (N675)*
*Alors pour la quantité, on leur demande à la fin du repas de bien écouter leur ventre, si elles en reveulent ou si c’est juste de la gourmandise, donc ça à force de répétitions je pense qu’elles ont compris la différence. (PRTAN01)*	*So for the quantity, we ask them at the end of the meal to listen to their bellies, if they want more or if it’s just greediness, so by hearing it time and again I think they have understood the difference. (PRTAN01)*
*Bon bah voilà elle adore ça, je ne vais pas lui dire non, non. […] Je veux vraiment que ça reste un plaisir de manger. Moi j’adore ça, j’adore ça manger, j’adore ça cuisiner et je veux vraiment que ce soit pour elle un plaisir. (K122)*	*Well, she loves it, I’m not going to say no to her, no. […] I really want it to remain a pleasure to eat. Me, I love it, I love to eat, I love to cook and I really want it to be a pleasure for her. (K122)*
